# Therapeutic Potential of Various Plant-Based Fibers to Improve Energy Homeostasis via the Gut Microbiota

**DOI:** 10.3390/nu13103470

**Published:** 2021-09-29

**Authors:** Taylor M. Martinez, Rachel K. Meyer, Frank A. Duca

**Affiliations:** 1 Physiological Sciences Graduate Interdisciplinary Program, University of Arizona, Tucson, AZ 85721, USA; taylormartinez@email.arizona.edu; 2 School of Nutritional Sciences and Wellness, University of Arizona, Tucson, AZ 85721, USA; rachelmeyer@email.arizona.edu; 3 School of Animal and Comparative Biomedical Sciences, University of Arizona, Tucson, AZ 85721, USA; 4 BIO5 Institute, University of Arizona, Tucson, AZ 85721, USA

**Keywords:** obesity, gut microbiome, dietary fiber, energy homeostasis

## Abstract

Obesity is due in part to increased consumption of a Western diet that is low in dietary fiber. Conversely, an increase in fiber supplementation to a diet can have various beneficial effects on metabolic homeostasis including weight loss and reduced adiposity. Fibers are extremely diverse in source and composition, such as high-amylose maize, β-glucan, wheat fiber, pectin, inulin-type fructans, and soluble corn fiber. Despite the heterogeneity of dietary fiber, most have been shown to play a role in alleviating obesity-related health issues, mainly by targeting and utilizing the properties of the gut microbiome. Reductions in body weight, adiposity, food intake, and markers of inflammation have all been reported with the consumption of various fibers, making them a promising treatment option for the obesity epidemic. This review will highlight the current findings on different plant-based fibers as a therapeutic dietary supplement to improve energy homeostasis via mechanisms of gut microbiota.

## 1. Introduction

Obesity is a growing epidemic affecting over 500 million adults globally. Global prevalence rates have tripled in the last 40 years, and about $100 billion is spent on obesity-related healthcare costs in the U.S. alone [1]. Obesity, in simple terms, arises from an imbalance between energy intake and energy expenditure. Given that estimates suggest expenditure rates have not significantly changed over the last few decades, it is more likely that obesity is resultant from an increase in the consumption of a highly palatable, calorically dense Western diet [2]. In the United States, obesity rates are positively associated with fat and sugar consumption, which feature prominently in a Western diet [2]. However, another characteristic of a Western diet is a low amount of fiber, or nondigestible carbohydrates. Accordingly, fiber intake is negatively associated with obesity [3], and increased fiber intake can improve metabolic outcomes in humans. Dietary fiber is metabolized by bacteria inhabiting the gastrointestinal tract, thus linking the gut microbiome with metabolic disease.

There is a burgeoning appreciation for the role of the gut microbiome in the development of obesity [3]. For example, obese rodents and persons with obesity exhibit a distinct gut microbiome that differs in composition, diversity, and functionality [4]. Additionally, inoculation of germ-free (GF) animals with an obese gut microbiota recapitulates the host phenotype [4], demonstrating the functional capacity of the gut microbiome on host metabolism. Therefore, there is an increased interest in targeting the gut microbiome for treatment and prevention of obesity. One way to alter the microbiome is through increased dietary fiber intake. Dietary fiber is described by the FDA as a carbohydrate component of an edible plant that is resistant to digestion and absorption [3]. While dietary fibers cannot be digested directly by the host, they can be fermented by gut bacteria in the distal intestine, resulting in the production of short chain fatty acids (SCFAs), which are known to improve energy homeostasis and metabolism [4]. 

**Figure 1:**
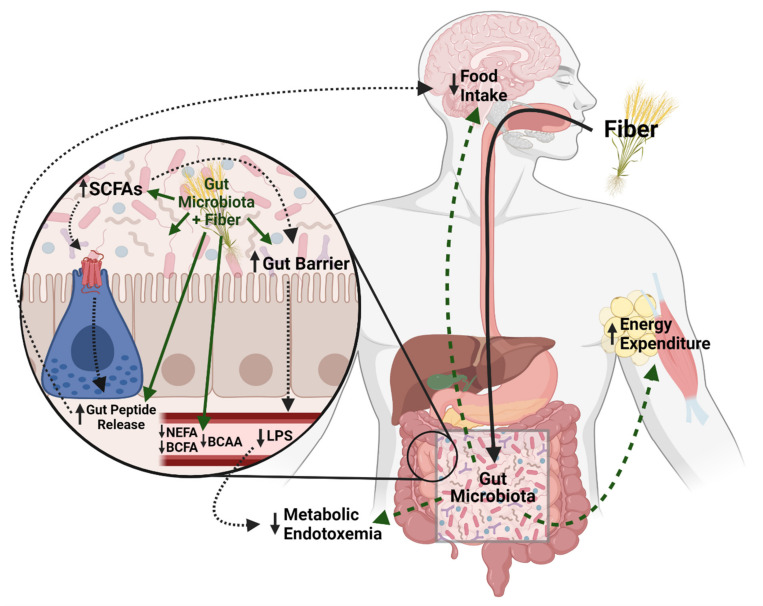
Potential Mechanisms for the beneficial effects of dietary fiber on metabolic homeostasis. Dietary fiber can shift the gut microbiota to promote gut barrier health and decrease circulating lipopolysaccharide (LPS) and subsequently lower metabolic endotoxemia. The gut microbiota also breaks down fiber into short chain fatty acids that can induce gut peptides that influence metabolic homeostasis, as well as impact the gut barrier. Additionally, dietary fiber can lower circulating non-esterified fatty acids NEFA), branched-chain fatty acids (BCFA), branched-chain amino acids (BCAA) which are associated with metabolic dysregulation. Dietary fiber may also increase energy expenditure although the mechanisms are not well understood. Created with BioRender.com (accessed on 9 August 2021).

Outlined in Figure 1 and detailed throughout the specific dietary fiber sections below, there are several main hypotheses as to how dietary fibers can improve host metabolic homeostasis. First, dietary fiber can shift the gut microbiome, promoting growth of more beneficial bacteria and reducing bacteria associated with metabolic disease [5,6,7,8]. Shifts in beneficial taxa, such as some *Bifidobacterium* and *Lactobacillus*, are associated with improvements in gut barrier [9,10]. Obesity and high-fat feeding are associated with increased gut permeability and increased translocation of lipopolysaccharide (LPS), a component of the cell wall of Gram-negative bacteria. Increased circulating LPS, coined metabolic endotoxemia, results in increased systemic inflammation via downstream LPS action on Toll-like receptor 4 signaling pathways [11]. Various dietary fibers have been shown to reduce endotoxemia by improving the gut barrier, possibly via increased beneficial bacteria, as well as increased production of SCFAs. For a more detailed review on the gut microbiome, metabolic syndrome, and metabolic endotoxemia, see Régnier et al., 2021 [12].

As mentioned above, most fiber can be fermented by the bacteria in the distal gut, producing SCFAs, mainly acetate, propionate, and butyrate. Early studies on the gut microbiome and metabolic disease hypothesized that SCFA production resulted in weight gain due to increased energy harvest from the diet. However, recent evidence suggests that SCFAs are beneficial to host health, which has been reviewed extensively elsewhere [13]. Briefly, SCFAs can act either as a fuel source, signaling molecules, or potentially epigenetic regulators [4]. Colonocytes predominantly utilize butyrate as a fuel source, which can promote gut barrier health and integrity [14]. SCFAs can also act as ligands, binding to G-coupled protein receptors, specifically free fatty acid receptor 2 (FFAR2) and free fatty acid receptor 3 (FFAR3) [15,16]. These receptors are localized on various metabolic tissues, including adipose, liver, neurons, and locally on enteroendocrine cells (EECs). Activation of distal EECs via SCFAs leads to secretion of gut peptides, mainly glucagon-like peptide-1 (GLP-1) and peptide YY (PYY), which can improve energy and glucose homeostasis via both endocrine and paracrine effects on vagal afferent neurons [16,17,18]. Lastly, SCFAs, especially butyrate, act as histone deacetylase inhibitors, thus promoting epigenetic changes; however, this mechanism is still being established [19,20]. In addition to improved gut integrity, reduced inflammation, and increased gut peptide signaling, dietary fiber could improve metabolic homeostasis via changes in energy expenditure, possibly mediated by improved thermogenesis or increased substrate oxidation [3,21,22]; however, this remains to be thoroughly explored.

Although this review primarily focuses on SCFAs, given they are direct metabolic byproducts of dietary fiber fermentation, it is important to note that several other metabolic pathways and specific metabolite intermediates are impacted by fiber supplementation, given that dietary fiber results in overall shifts in the gut microbiome. For example, mice colonized with human fecal samples fed a diet containing 10% *w*/*w* cellulose, inulin, pectin, or a mix of 5 fermentable fibers for 4 weeks exhibited changes in serum metabolites from amino acid (including branched chain amino acid), fatty acid, endocannabinoid, and sphingolipid metabolic pathways [23]. Interestingly, serum sphingomyelins were increased primarily in mice fed the pectin diet, which was associated with increased adiposity. Further, serum histidine was found to be negatively correlated with glucose and adiposity, and was increased in mice supplemented with inulin in the same study [23]. Given that histidine supplementation attenuates inflammatory markers [24] and decreases food intake and adiposity [25] in obese rats, this metabolite may, at least partially, mediate the beneficial effect of fiber supplementation on systemic inflammation. Given the vast number of microbially derived metabolites, dietary fiber likely impacts production of many metabolites influencing host health; however, the literature on this topic is limited. Future studies investigating the effect of individual fiber types on microbial metabolites is therefore necessary to gain a complete understanding of their contribution to host energy and glucose homeostasis, and this review will focus on more extensively studied metabolites such as SCFAs and branched chain amino acids (BCAAs).

While the current review focuses on the role of the gut microbiome, it is also important to note that dietary fiber could alter intestinal nutrient absorption. For example, highly viscous, soluble fiber forms a gel in the intestines, physically blocking fat and other nutrients from digestion and absorption and decreasing the energy available to harvest from nutrients [26,27]. However, more work is needed to delineate the effects of the physical properties of dietary fiber on host metabolism from the effects of the gut microbiome and metabolites. Taken together, there are many proposed mechanisms that are likely involved in the ability of dietary fiber to improve metabolic homeostasis (Figure 1).

Dietary fibers can be found in fruits, vegetables, grains, and fungi. In addition to natural sources, many of these fibers are commercially available in supplemented cereals or prebiotic drink mixes. Although increased overall fiber consumption improves obesity, dietary fibers differ in composition, which likely affects the therapeutic potential of specific fibers on body weight and metabolic parameters. For example, the primary differences in fiber composition affect the fiber solubility and viscosity, which determine how easily the fiber is fermented by gut bacteria and transit time through the intestinal tract [28,29]. It is hypothesized that highly soluble fibers are more easily broken down by bacteria in the gut, while higher viscosity fibers stay in the intestinal tract longer to maximize that fermentation [28,29]. Given the heterogeneity of plant-based fibers, it is imperative to better understand the beneficial uniqueness of specific fibers in relation to host metabolic homeostasis. Therefore, the following review will explore the metabolic impact of some of the most studied dietary fibers in regard to energy homeostasis. Specifically, this review will detail the host metabolic effects of high-amylose maize, β-glucan, wheat fiber, pectin, inulin-type fructans, and soluble corn fiber which differ in their main properties and natural sources (Table 1). A better understanding of the contribution of a dietary fiber to alter energy intake and expenditure could lead to better treatment options for obesity and other metabolic-related conditions.

## 2. High-Amylose Maize

High-amylose maize is a type of resistant starch, which has been extensively researched for its efficacy as a treatment for obesity [30,31]. Resistant starch receives its name for the ability to travel through the small intestine without digestion, due to being composed mainly of insoluble granules that are undigestible by amylase. This occurs in contrast to normal dietary starch that is hydrolyzed in the brush border of the intestinal epithelium. As such, resistant starch reaches the distal intestine, where it is fermented to produce SCFAs [32]. In a recent meta-analysis, it was determined that resistant starch supplementation improved insulin sensitivity and lowered blood glucose and fasting insulin levels in patients with type 2 diabetes and obesity [33]. The authors speculated these metabolic improvements were due to modulation of gut bacterial composition and increased SCFA production [33]. There are 5 different classifications of resistant starch, differing in composition, preparation, and fiber content, with type 2 being most commonly studied for modulation of energy and glucose homeostasis. Type 2 resistant starch, and, more specifically, high-amylose maize, has been demonstrated to reduce adiposity, increase colonic SCFAs, and increase gut peptide secretion in rodents [30,31,34,35].

Low-fat diets supplemented with 28% high-amylose maize in place of cellulose for 12 weeks resulted in decreased abdominal fat in male rats that was associated with increased serum GLP-1 and PYY levels, in addition to an altered gut microbiota [31]. Despite increased GLP-1 and PYY, both known to increase satiety and satiation, there was no difference in food intake between the groups, confounding the potential mechanism for the observed beneficial effect. A similar dose of high-amylose maize (29.7%) in low-fat diet decreased body weight and altered the gut microbiota in female sham and ovariectomized rats, despite actually increasing food intake [30]. However, supplementation for 12 weeks with 27% high-amylose maize in HF-feeding did not reduce abdominal fat in rats, despite slightly increasing SCFAs, albeit not to the extent observed in low-fat fed rats [36]. Thus, the degree of SCFA production may be key to the success of high-amylose maize. A similar study in high fat diet (HFD)-fed mice supplemented with 20% high-amylose maize for 6 weeks found no difference in adiposity or body weight, and supplemented mice exhibited increased food intake compared to controls [37]. These inconsistencies may be due to the study duration of the fiber-supplemented diet, and highlights that the dietary fiber may require more time for beneficial results to be seen; alternatively, it suggests that high-amylose maize may only be beneficial as a preventative agent for obesity and not as a therapeutic to improve dysregulated metabolic homeostasis. Studies detailing no change or increases in food intake, but no change in body weight, suggest high-amylose maize could be increasing energy expenditure, although this remains to be assessed.

Several, but not all, studies have demonstrated that high-amylose maize supplementation specifically increases butyrate levels with no change in other SCFAs. A chow diet composed of 28% high-amylose maize increased cecal butyrate in rats, while high protein-fed rats given 10% high-amylose maize exhibited the same result [31,38]; however, HFD-fed rats supplemented with 20% high-amylose maize had no differences in SCFA concentrations compared to controls [37], again highlighting the fact that inability to produce substantial amounts of SCFAs is key to the beneficial metabolic effect of high-amylose maize. Butyrate is known to induce GLP-1 and PYY release from EECs and increase gut peptide mRNA expression both in vitro and in vivo [31,39,40]. As such, both low fat and high fat diets supplemented with varying concentrations of high-amylose maize result in increased circulating and gene expression levels of GLP-1 and PYY [31,36,37]. However, given there were no effects on food intake or glucose homeostasis with high-amylose maize supplementation, the contribution of these improvements in gut peptide signaling on overall metabolic homeostasis is confounding. Thus, improvements in energy homeostasis might be due to different mechanisms. For example, given its ability to be fermented, it is not surprising that high-amylose maize alters the gut microbiota and metabolome. In fact, HFD-fed, high protein diet-fed, and low fat diet-fed rodents supplemented with varying concentrations of high-amylose maize all demonstrated distinct gut microbiota profiles with decreased bacterial diversity compared to controls [31,37,38]. Interestingly, both 20% high-amylose maize in HFD and 10% high-amylose maize in a high-protein diet, with no change to the protein content of each diet, reduced serum branch chain amino acids (BCAAs) [37,38] in rodents. Circulating BCAAs are increased in subjects with metabolic disease and are becoming increasingly recognized as a biomarker for obesity and diabetes [37]. Thus, changes in circulating BCAAs via high-amylose maize could be contributing to its beneficial effect in specific studies.

Unfortunately, human trials with high-amylose maize supplementation have produced mixed and confounding results. On the one hand, 4 weeks of high-amylose maize supplementation (30 g/day) improved insulin sensitivity (HOMA%S, *p* = 0.0008, treatment condition *p* = 0.018, treatment × sex interaction *p =* 0.033) in healthy individuals [41]. Further, 57 days of high-amylose maize supplementation also decreased fasting (22%, *p* = 0.04), 2 h postprandial (23.3%, *p* < 0.008), and 3 h postprandial (18.9%, *p* = 0.05) plasma insulin and improved HOMA-IR (23.1%, *p* = 0.04) in individuals with overweight/obesity with an increased risk for type 2 diabetes [42]. On the other hand, investigators have found that patients diagnosed with type 2 diabetes and metabolic syndrome exhibited no improvements in fasting glucose, fasting insulin, or energy intake, despite increased GLP-1 (11.4 ± 1.9 vs. 17.0 ± 3.2, *p* = 0.049), with 40 g/day of high-amylose maize consumption [43]. However, this study demonstrated that circulating non-esterified fatty acids (NEFAs) decreased with supplementation (500 ± 100 vs. 600 ± 50, *p* = 0.004) [43]. A similar study supplementing 20.7 g resistant starch (as a mixture of high-amylose maize and arabinoxylan) found that fecal branched chain fatty acids (BCFAs) decreased by 30% (*p* = 0.03) [44]. Elevated circulating NEFAs are associated with increased adipose tissue [43], and the authors hypothesize decreased NEFAs signify stimulation of adipose FFAR2 and FFAR3 by microbial fermentation products [43]. Additionally, a decrease in fecal BCFAs is indicative of diminished protein fermentation, possibly due to observed reduction in *Bacteroides* abundance, a bacterial genus containing species responsible for BCFA production [44]. Thus, decreases in circulating BCAA and NEFA and fecal BCFA could represent a potential beneficial effect of high-amylose maize on obesity and energy homeostasis. Taken together, the research on high-amylose maize demonstrates a potential beneficial effect of supplementation on energy homeostasis and metabolism, at least in healthy subjects; however, many gaps in this area of research still remain, including the causative effect of alterations to the gut microbiome, changes in adiposity, and whether increase in circulating gut peptides occur via increased SCFAs and are causal to weight loss.

## 3. β-Glucan

β-glucan is a non-digestible dietary fiber found in many food sources including barley, oat, and yeast products [45]. β-glucans are found in the cell wall of an endosperm and are classified as glucose monomers connected by beta glycosidic bonds [46]. β-glucan is further distinguished by high viscosity and water solubility, which have been demonstrated to lengthen transit time through the small and large intestine and increase fermentation by gut bacteria, respectively, making β-glucan supplementation an area of interest for obesity and metabolic disorder research [29]. Overall, β-glucan as a dietary supplement has been suggested to decrease body weight and regulate glucose homeostasis in both human and animal studies. While β-glucan isolated from various sources has shown positive, though variable, effects, oat and barley products appear to have the strongest potential for treating obesity. For example, treatment with a barley-rich diet containing either 4.4 g/day β-glucan over 12 weeks in humans with obesity resulted in a decrease in body mass index (25.9 ± 2.9 vs. 26.2 ± 2.8, *p* < 0.001) and visceral fat area (91.7 ± 36.8 vs. 102.3 ± 41.2, *p* < 0.01) compared to baseline [47]. A similar study supplemented 7 g/day β-glucan for 12 weeks and resulted in significant reductions in body weight and BMI in the β-glucan group (−0.4 kg body weight and −0.2 kg/m^2^ BMI with intervention, *p* = 0.004 and *p* = 0.005, respectively) [48]. However, it is important to note that the diets contained other barley ingredients besides β-glucan, so changes in micronutrients could have had an impact [47,48]. In HFD-fed mice, 8.5% oat β-glucan supplementation for 8 weeks resulted in decreased body weight and improved insulin sensitivity and HOMA-IR [49], while diabetic rats fed a chow diet high in barley flour with 6% β-glucan for 6 weeks exhibited decreased food intake and decreased blood glucose compared to diabetic controls [50]. Furthermore, a human study reported decreased feelings of hunger after oat bran supplementation with 1.6 g β-glucan per day that was linked with increased viscosity of the oat bran meal, suggesting that the more viscous oat-supplemented foods lead to slower gastric emptying and further decreased food intake and increased satiety [28]. Interestingly, decreased food intake and blood glucose observed following a high barley flour diet containing 6% β-glucan in diabetic rats was also attributed to an increase in food viscosity [50].

In addition to viscosity, many studies suggest that the effects of β-glucan supplementation, whether isolated or via a flour, may be due to alterations in the gut microbiota that promote increased SCFA production [4]. Improved energy and glucose homeostasis in HFD-induced obese mice with barley supplementation containing 4% β-glucan was associated with an increase in gut *Actinobacteria*, a phylum known to increase SCFA production, and increases in fecal acetate, butyrate, and propionate [51,52]. As mentioned previously, SCFAs are known to increase secretion of gut peptides, and accordingly, plasma PYY and GLP-1 levels were increased in this study [52]. Interestingly, GF mice fed the same diet had no change in body weight, PYY, or GLP-1 levels, suggesting the gut microbiome is necessary to see these beneficial changes [52]. However, GF mice are known to be resistant to HFD-induced obesity and have relatively high circulating GLP-1 levels [52], thus their inherent metabolic differences may have masked an effect. Nonetheless, 5% β-glucan supplementation in HFD-fed mice resulted in decreased body weight, and increased fecal SCFA and *Actinobacteria* [52]. However, at least one study argues that the gut microbiota is not necessary for improvements observed with barley supplementation, and, in fact, the gut microbiota may lessen the beneficial impact [53]. Gong et al. observed an increase in adiposity and fecal SCFA production in humanized gut microbiota mice compared to GF mice both fed a HFD supplemented with 46% whole barley [53]. While the authors suggest that increased adiposity occurred due to increased energy harvest from the diet, the results are difficult to interpret given that the diets were not completely ingredient, macronutrient, or calorically matched. Furthermore, the inoculation was carried out from the feces of only one human donor, and recent work has found that the human microbiome exhibits ‘personalized’ responses to various diets and fibers [54]. While the humanized mice should have increased SCFAs compared to GF, given the gut microbiota is necessary for SCFA production, it does not prove causality of increased adiposity as conventionalization of GF mice results in many physiological, metabolic, immune, and neural changes [23]. Furthermore, there was no HFD control to determine if, despite increased adiposity in humanized mice fed a HFD-whole barley diet compared to GF, mice fed a HFD-whole barley diet had decreased adiposity compared to HFD alone [53].

Many studies show that β-glucan supplementation results in weight and fat loss with a concurrent reduction in food intake. For example, in humans, acute 3 g β-glucan supplementation decreased hunger by 49% (−5761 ± 2944 vs. −3863 ± 2312, *p* < 0.05), increased satiety by 55% (3444 ± 1980 vs. 2221 ± 1375, *p* < 0.05) and decreased energy intake by 19% (−172 ± 8.5 kcal, *p* < 0.05), which was associated with decreased plasma ghrelin and increased PYY [55]. HFD-fed rodents supplemented with barley flour containing varying amounts of β-glucan exhibit reduced fat mass, body weight, cholesterol and increased insulin sensitivity, with some, but not all, studies finding a reduction in food intake [21,45,56,57]. Although not all studies directly show a decrease in food intake, given the rise in SCFA production and increase in the circulating gut peptides, PYY and GLP-1 observed following β-glucan supplementation [21,39,40,47,48,56], it is possible that β-glucan supplementation increases gut-brain signaling that regulates metabolic homeostasis. For example, in HFD-induced obese rats, oat flour supplementation contributing 7% β-glucan in a normal chow diet decreased food intake, body weight, and adiposity that was associated with an increase in plasma PYY [57]. This rise in PYY was associated with suppression of arcuate nucleus NPY mRNA, which is known to regulate food intake and energy homeostasis [57]. One study, however, found that rats on a chow diet supplemented with oat flour containing 1.6% β-glucan exhibited increased cecal butyrate and decreased fat-pad weight in the face of increased food intake [58]. Thus, most animal and human studies suggest that improvements in energy homeostasis via β-glucan supplementation could occur due to reductions in food intake, but a lack of consistent findings raises the possibility that β-glucan can alter energy expenditure, although this remains to be accurately assessed. Along those lines, UCP1 expression is increased after barley flour supplementation, suggesting that white adipose tissue browning may play a role in weight loss [21].

In addition to increasing SCFA production, beneficial shifts in the gut microbiota are commonly associated with improvements in gut barrier and subsequent metabolic endotoxemia and systemic low-grade inflammation [45]. As such, reductions in body weight in mice on HFD with barley supplementation have been associated with replenished mucosal thickness, colonic length, and goblet cell numbers [46]. One study found β-glucan supplementation (3 or 5 g/kg body weight) resulted in an increase in *Mucispirillum*, a bacterium that feeds on mucin, which they suggest is associated with increased mucin production and a correlation with increased gut integrity resulting from the β-glucan [45]. This is in line with a human study that showed overweight males given 477 mg/day β-glucan for 6 weeks had a decrease in waist circumference (86.5 ± 8.9 cm vs. 94.69 ± 3.32 cm, *p* = 0.037) and blood pressure (−5.32 mmHg, *p* = 0.035) that was associated with a modulatory effect on inflammation, specifically a 31.12% increase in interleukin-10 (IL-10, *p* < 0.001 compared to baseline and control group), an anti-inflammatory cytokine, and a reduction in the proinflammatory cytokines, interleukin-6 (IL-6, *p* = 0.005 compared to control group) and tumor necrosis factor-alpha (TNF-alpha, *p* = 0.037 compared to control group) 6 and 2 weeks into treatment, respectively [59]. Overall, β-glucan displays promising results to decrease body weight and adiposity in both a fiber form as well as a main component in barley and oat flour. However, more research is needed to identify possible explanations for these effects and whether they arise from alterations to the gut microbiome, changes in energy expenditure, or by affecting intestinal integrity.

## 4. Wheat Fiber

Fiber derived from wheat is commonly studied for its effects on energy and glucose homeostasis, given its high commercial availability and ability to be easily obtained in large quantities [60]. The wheat bran fraction is the outer layering and embryo of wheat grain that is separated from the endosperm and processed for palatability [61]. It is a source of insoluble fiber that is milled into a flour commonly used in breads and cereals [61]. Though most fiber studies suggest soluble fiber is more beneficial in reducing obesity due to high fermentability, insoluble fibers may provide favorable changes to energy homeostasis. In rodents, wheat bran supplementation (0.8–5%) attenuates HFD-induced weight gain and decreases adiposity [62,63], while in humans, acute supplementation of 41 g wheat bran decreased subsequent energy intake (*p* = 0.02) [64]. Wheat bran supplementation has also been associated with improvements in glucose homeostasis, as evidenced by decreased serum glucose and HOMA-IR in rodent models [62,65,66,67]. Additionally, wheat bran supplementation is associated with other positive health benefits such as decreased serum lipids and free fatty acids and improved HDL in rodents [62,65,67]. Interestingly, despite decreased body weight in rodents following wheat bran supplementation, no studies observed a significant decrease in food intake, with one study actually displaying an increase in food intake with as low as 0.8% dietary wheat bran supplementation in HFD for 24 weeks [62]. However, these mice exhibited an increase in physical activity compared to calorie-matched HFD-fed mice [62], possibly indicating increased energy expenditure driving weight loss. 

Regardless, wheat bran supplementation alters the gut microbiota, possibly leading to improved gut integrity and reduced inflammation. Mice on a HFD supplemented with 5% wheat bran for 8 weeks had increased richness in microbiome diversity in addition to an increase in *Akkermansia muciniphilia* and *Bifidobacterium* abundance. *Akkermansia muciniphilia* specifically has been associated with attenuated weight gain [68], and *Bifidobacterium* species, including *Bifidobacterium breve*, are known to prevent fat accumulation [69]. Further, supplementation of wheat bran at 7.5% for 8 weeks reduced body weight and adiposity, which was associated with a decrease in *Lactobacillus* cecal abundance [70]. In line with these bacterial shifts, wheat bran supplementation is associated with a decline in gut inflammation, with reductions in the inflammatory cytokines TNF-alpha and IL-6 [60,62], and increased tight junction proteins that are associated with reduced endotoxemia and anti-inflammatory cytokines [66]. Interestingly, mice on a HFD supplemented with 10% wheat bran for only 3 weeks exhibited an increase in cecal *Lactobacillus*, despite no changes in body weight or adiposity [60]. While these studies show a possible association between *Lactobacillus* abundance and weight/adiposity, it is important to note that not all *Lactobacillus* species are beneficial and more research into strain specific alterations is necessary. Overall, these results suggest that wheat bran supplementation can alter the gut microbiome and may improve energy and glucose homeostasis; however, more human research is necessary to confirm the beneficial health outcomes of wheat bran supplementation.

Wheat dextrin is a soluble fiber commonly used as a powder supplement in fluids due to its low viscosity [71]. Although less studied, wheat dextrin supplementation (10 g/day) for 8 weeks decreased body weight (−3.1 kg, *p* < 0.05), BMI (−1.4 mg/kg^2^, *p* < 0.05), fasting insulin (−21.17 pmol/L, *p* < 0.05), and HOMA-IR (−1.55 AU, *p* < 0.05) in humans with type 2 diabetes [72]. This study also found wheat dextrin reduced systemic inflammation, with decreased circulating IL-6 (−1.4 pg/mL, *p* < 0.05), TNF-alpha (−2.3 pg/mL, *p* < 0.05), MDA (−1.10 nmol/mL, *p* < 0.05), and LPS (−4.4 EU/mL, *p* < 0.05) [72], similar to wheat bran. Although the mechanism of action remains largely unknown, studies have linked wheat dextrin with increased SCFA production [73,74]. In vitro human fecal samples in an anaerobic chamber, used to replicate human intestinal conditions, resulted in significantly increased SCFA production, mostly acetate, when supplemented with a 1% wheat dextrin medium [73]. This study also found a large shift in the gut bacterial community, with decreased diversity and increased abundance of *Bacteroides* and *Parabacteroides*, bacteria known to digest resistant starches and complex carbohydrates [73]. In humans, supplementation with increasing doses (10, 15, and 20 g per day) of wheat dextrin for 2 weeks increased fecal *Bacteroides* abundance and modestly increased SCFA production, although the increase in SCFAs did not reach statistical significance [75]. Additionally, 20 g per day of wheat dextrin supplementation decreased colonic and fecal pH, indicating an increase in fermentation, and was correlated with a decrease in possibly harmful bacteria [73,75]. Overall, wheat dextrin and wheat bran show promising results for altering the gut microbiota to produce potential benefits in obesity, such as decreased body weight, systemic inflammation, and serum cholesterol; however, more research on the therapeutic effect of wheat fiber is needed in diet-induced obese animal and human studies.

## 5. Pectin

Pectin is a soluble dietary fiber found in the cell wall of many fruits and vegetables [76]. Similar to the other fibers discussed in this review, pectin is unable to be digested by the host but can be fermented by gut microbiota. However, the lower viscosity of pectin relative to β-glucan may result in different effects observed in individuals with obesity or obese animals [76]. Pectin supplementation (5–10% *w*/*w*) to both chow and HFD-fed rodents decreases weight gain, adiposity, and food intake [77,78,79]. Interestingly, switching diet-induced obese mice to 10% *w*/*w* pectin supplementation HFD for 5 weeks prevented weight gain without decreasing food intake [80]. These findings have been replicated in humans with metabolic syndrome, as pectin supplementation for 90 days decreased body weight by 14.8% (*p* < 0.05) and BMI by 15.9% (*p* < 0.0001) [81]. Further, these individuals had a 18% decrease in fasting glucose (*p* < 0.01) and an 18.1% decrease in HOMA-IR (*p* < 0.001) [81], indicating a potential for pectin as an anti-hyperglycemia therapy for individuals with type 2 diabetes. This effect has also been observed in humans with overt type 2 diabetes who had improved glucose tolerance in a mixed meal test (27.9 ± 3.2 vs. 34.8 ± 3.0 mmol/L, *p* < 0.01) after 4 weeks of 20 g per day of pectin supplementation [82]. Similar effects of pectin on glucose homeostasis have been observed in rodent studies, with pectin supplementation reducing plasma insulin [77,83] and blood glucose [80] at a dose of 10% *w*/*w* in HFD-fed rodents. 

As seen with β-glucan, soluble dietary fibers, including pectin, are digested by gut bacteria to produce cecal SCFAs that can induce gut peptide release and reduce food intake [77,78]. As such, varying concentrations of pectin supplementation in either HFD and chow diets increase both SCFA levels and circulating GLP-1 and PYY in rodents [77,78,84,85,86]. However, the increases in specific SCFAs differed between studies, with some displaying an increase in acetate and propionate and others only in butyrate [77,78,84,85,86]. This discrepancy may occur due to inconsistencies in diet macronutrient composition and ingredients, or the increase in distinct gut bacteria that correlate with production of only one type of SCFA. In vitro models using human fecal samples in a medium designed to replicate anaerobic large intestinal conditions also demonstrate increased SCFA production with pectin supplementation [5,6,73,76]. The majority of studies found the greatest increase in acetate [5,6,73], while only one study observed the greatest increase in propionate [76]. The differences in the SCFAs produced may also be due to the distinct changes in gut bacterial abundance observed with pectin supplementation. While an increase in *Bacteroides* abundance is the most common result of pectin supplementation, *Bifidobacterium* is also increased [6,73,83,86]. Each of these bacterial shifts is consistent with pectin degradation resulting in the production of various SCFA. Additionally, these bacteria are also associated with decreases in obesity-related gut inflammation [6,73,83,86]. Indeed, pectin supplementation (4–10% *w*/*w*) in rodent models of diet-induced obesity results in increased tight junction proteins and decreases in the inflammatory cytokines TNF-alpha, IL-6, and nuclear factor kappa B (NFκB) [79,83,85] while also increasing *Proteobacteria* [6]. Interestingly, many species of *Proteobacteria* are considered pathogenic and characteristic of gut microbial dysbiosis [87]; therefore, additional microbiome sequencing at the species level is crucial to elucidate specific *Proteobacteria* species abundance and involvement in host health.

In addition to the improvements seen in energy and glucose homeostasis, pectin treatment decreases circulating LDL while increasing HDL, and decreases triglycerides in the serum and liver, making it a promising treatment for metabolic syndrome [77,78,85,88]. Obese rodents fed a diet supplemented with 10% pectin exhibited decreased cecal BCFAs, which are associated with poor colon health [77], and hepatic NEFAs, which are associated with insulin resistance [85]. However, studies in healthy adult subjects demonstrated that pectin supplementation in water for 4 weeks resulted in no change in small intestine or colonic permeability [89] and had no effect on the gut microbiota composition or plasma SCFA concentrations [90]. This suggests that pectin may have limited effects on otherwise healthy humans, indicating that it would be beneficial as a treatment for metabolic disease. 

## 6. Inulin-Type Fructans

Fructans are found in many fruits and vegetables and are composed of beta-(2,1) fructosyl-fructose linkages. “Inulin-type fructan” is the generic term to cover all beta-(2,1) linear fructans that have a variety of health benefits. Inulin is mainly found in chicory root and has a beta glycosidic bond configuration that makes it resist hydrolysis from alpha specific enzymes in the intestinal tract [3]. Inulin and oligofructose are the two most researched inulin-type fructans in regard to energy homeostasis and glucose metabolism. When used as a long-term dietary fiber supplement in overweight humans, 20 g per day of inulin for 42 days decreased plasma insulin (9.0 ± 1.2 vs. 12.3 ± 1.4 µU/mL, *p* = 0.004) and improved insulin sensitivity (HOMA2-IR, *p* < 0.01) [7]. Further, an acute dose of 24 g inulin in men with obesity decreased postprandial (0–3 h) plasma glucose (glucose iAUC, 8.12 ± 3.07 vs. 46.97 ± 3.57 mmol/L, *p* = 0.002) and insulin (insulin iAUC, 1494 ± 81 vs. 3523 ± 161 mU/L, *p* = 0.001) while increasing fat oxidation (*p* < 0.05) [91]. In rodents, inulin supplementation at 10% *w*/*w* also leads to a decrease in body weight and fat mass [92], a result that was recapitulated in individuals with prediabetes given 30 g inulin daily for 18 weeks [93]. 

A differentiating characteristic of inulin and oligofructose is the degree of polymerization, with inulin having a longer polymer chain compared to oligofructose. Utilizing the SHIME system, a 5-vessel model containing bacterial communities used to replicate the human digestive tract, it was demonstrated that both inulin and oligofructose increase SCFA production, with higher concentrations of propionate and butyrate from inulin supplementation [94]. This finding, along with an increase in ammonium production from oligofructose, led the authors to conclude that the longer polymerized inulin was more effective and safer for consumption. Inulin supplementation in both human and animal studies has also shown to increase the abundance of *Bifidobacteria*, *Bacteroides*, and *Actinobacteria* [7,92,94,95,96,97] in the gut, some of which are known to be beneficial or associated with host health [69,70,98]. These microbial changes are hypothesized to lead to the observed increases in SCFA production [71,91]. However, not all studies have demonstrated that inulin supplementation increases SCFA levels [96], suggesting that more research may be needed to further elucidate the effect of inulin on SCFA production. 

Oligofructose is a non-digestible carbohydrate that, such as pectin, is soluble and non-viscous. The fructan is composed of short chain oligomers and is found in many fruits and vegetables such as bananas and onions [99]. Oligofructose supplementation attenuates HFD-induced weight gain and adiposity, which was associated with a decrease in food intake [8,51,100,101,102,103]. This effect was observed across studies, despite using varied doses and study duration (5% *w*/*w* for 8 weeks, 10% for 15 days-6 weeks, 0.3 g/day for 8 weeks) [8,51,100,101,102,103]. Several human studies recapitulate these findings. Oligofructose supplementation (~30 g/day) for as few as 30 days decreased body weight in children with genetic (−7.6 ± 0.6%) or simple (−9.5 ± 0.4%) obesity, improved fasting hyperglycemia (*p* < 0.01) and decreased oral glucose tolerance (OGTT glucose AUC, *p* < 0.01) after 60 days [104]. Another study in adults with overweight or obesity found that 21 g/day oligofructose supplementation for 12 weeks without changes in physical activity or lifestyle modifications significantly decreased body weight (−1.03 ± 0.43 kg, *p* < 0.05), fat mass (*p* = 0.005), and trunk fat (*p* = 0.05), without affecting glucose homeostasis [105,106]. However, not all human studies demonstrate successful weight loss, as 12 weeks of daily 16 g oligofructose supplementation resulted in no improvements in body weight or food intake; however, the investigators suggest this may have been due to decreased adherence to study instructions or a small sample size of subjects [107]. In regard to improvements in glucose metabolism, oligofructose treatment for as few as 4 weeks lowers blood glucose levels and improves glucose and insulin tolerance in HFD-fed rodents at varying supplemental doses [51,102,103]. Replacing simple sugar in yogurt beverages with oligofructose also results in lowered postprandial blood glucose (glucose iAUC, 31.9 ± 3.2 mmol/L/min vs. 37.3 ± 3.0 mmol/L/min, *p* = 0.02) and insulin (insulin iAUC, 1598.2 ± 115.0 µU/mL/min vs. 1924.9 ± 144.6 µU/mL/min, *p* = 0.007) in healthy male and female adults without any change in taste, making it an attractive replacement of high-glycemic sugars [108]. One potential mechanism for the beneficial effects of OFS could be via reductions in obesity-associated gut permeability and low grade endotoxemia. Oligofructose treatment has been shown to decrease inflammatory cytokines in children with Celiac disease [109] as well as decrease LPS and interleukin-1 (IL-1) in genetically obese mice [51]. Additionally, similar to other fibers, oligofructose increases SCFA production and gut peptide signaling. Acetate, propionate, and butyrate are increased in human fecal samples after oligofructose treatment [109,110]. Circulating GLP-1 and PYY are also increased following oligofructose treatment in both human and animal studies [100,105,111], and oligofructose fails to improve adiposity and glucose tolerance in GLP-1 receptor knockout mice or mice with chronic GLP-1R antagonism [112].

These potential mechanisms driving the beneficial effects of oligofructose may be due to upstream alterations of the gut microbiome, as has been reviewed extensively elsewhere [8,51,100,108,109,110]. Oligofructose supplementation for 90 days resulted in weight loss in children with genetic (−7.6 ± 0.6%) or simple (−9.5 ± 0.4%) obesity that was associated with a decrease in bacterial diversity and an increase in *Bacteroides* and *Bifidobacterium* abundance, suggesting increased carbohydrate digestion and the production of SCFAs [104]. These shifts were further demonstrated to be causal for body weight effects, as inoculation of GF mice with post-oligofructose treatment gut microbiota results in less weight gain compared to pretreatment gut microbiota. Rats fed a HFD with oligofructose had an increased abundance of *Bifidobacterium*, *Lactobacillus*, and *Actinobacteria* [8,100] in the cecum while *ob/ob* mice treated with the same diet also had increased *Bifidobacterium* and *Actinobacteria*, which have both been reported to be negatively associated with obesity [113,114], as well as *Proteobacteria* [51]. However, antibiotic treatment prevented increases in the abundance of *Bifidobacterium* and *Lactobacillus* from OFS treatment, and, as such, abrogated the decreased adiposity from oligofructose treatment [100]. These observations, coupled with the fact that the physiological effects of OFS treatment are not observed in GF rats [115], highlight the fact that the beneficial effects of oligofructose are dependent on shifts in the gut microbiota. Overall, oligofructose is one of the most promising fiber supplements to improve metabolic parameters in humans.

## 7. Soluble Corn Fiber

Soluble corn fiber is a maize-based prebiotic obtained from corn starch and is another glucose polymer composed of glycosidic linkages [116]. Because of the fiber’s high solubility, it has high digestive tolerance when added to foods without inflicting unpleasant gastrointestinal symptoms, making it a promising dietary supplement [117]. While research examining the effects of soluble corn fiber on energy homeostasis is limited, some studies have investigated how soluble corn fiber interacts with calcium absorption and glycemic control [116,117,118,119]. Additionally, supplementation with a 10 or 20 g fiber supplement consisting of 85% soluble corn fiber daily for 4 weeks results in changes to the gut microbiota, especially in taxa correlated with bacteria known to digest starch and produce SCFA [118]. In HFD-fed mice, 10% soluble corn fiber supplementation in drinking water decreased body weight, fat mass, and improved glucose tolerance [120]. Similarly, administration of a test meal containing 26 g soluble corn fiber to healthy males attenuated the postprandial glucose response by 20% (glucose iAUC following a test meal, *p* < 0.05) and insulin response by 40% (insulin iAUC following a test meal, *p* < 0.001) compared to control meals [116]. However, a single test meal supplemented with 54.6 g soluble corn fiber did not significantly alter plasma glucose in overweight adults, but did attenuate the postprandial insulin response (*p* = 0.001) [22]. This may demonstrate that long-term soluble corn fiber supplementation is needed to see the full benefits of this fiber on glycemic control.

Due to the prebiotic characteristics, it has been well documented that soluble corn fiber causes beneficial changes to gut microbiota. Healthy adolescent females on a soluble corn fiber supplemented diet (10 or 20 g daily) for 4 weeks had a dose-dependent increase in *Parabacteroides* and *Bifidobacterium* abundance [118], while healthy elderly adults had an increase in *Parabacteroides* abundance after 3 weeks of 6 g daily soluble corn fiber supplementation [121]. Piglets born to sows on 2% soluble corn fiber supplemented food during gestation and nursing had increased *Bacteroides*, *Lactobacillus*, and *Actinobacteria* abundance in feces, in addition to higher weight at the end of the study and increased weight gain from birth [122]. Mice on a HFD supplemented with soluble corn fiber for 8 weeks surprisingly demonstrated a decrease in *Proteobacteria*, a phylum often negatively associated with obesity, but still had an increase in *Firmicutes* and counteracted the HFD-induced increase in obesity-related phyla such as *Ruminococcus, Bilophila, Desulfovibrio, Oscillospira* and *Paenibacillus* [120]. The digestion of this fiber by gut bacteria, similar to many of the other fibers, has been shown to produce SCFAs. An increase in total fecal SCFAs in humans was seen after one week of supplementation, while increased fecal and plasma acetate, butyrate, and total SCFAs was seen in piglets nursing from soluble corn fiber fed mothers [122,123].

Similar to other fibers, increased soluble corn fiber consumption reduces intestinal inflammation [120,122]. Piglets nursing from soluble corn fiber-fed mothers had decreased endotoxin and an increase in the anti-inflammatory mediator IL-10 and immune tolerance mediator transforming growth factor-β [122]. Additionally, HFD-fed mice supplemented with 10% soluble corn fiber had decreased inflammatory cytokines, such as monocyte chemoattractant protein 1, and trended towards decreased TNF-alpha and IL-6 [120]. Similarly, a colitis mouse model on a soluble corn fiber diet for 47 days exhibited reduced disease severity, which was associated with increased peroxisome proliferator activated receptor-alpha and suppressor of cytokine signaling 3 mRNA expression, both of which may have an anti-inflammatory and gut permeability effect [124]. In the single study that examined energy homeostasis, 10% soluble corn fiber in HFD decreased fat mass and adipose depots in mice and prevented HFD-induced weight gain without a change in energy consumption, suggesting the benefits may be independent of caloric consumption [120]. Other benefits of soluble corn fiber supplementation are increased fat oxidation with increased energy expenditure [22], decreased stool pH [118,123] and increased calcium absorption [118,119]. While the research on soluble corn fiber is more limited than the previously reviewed fibers, it remains a promising fiber to address HFD-induced changes in energy homeostasis.

## 8. Conclusions

Dietary fibers can be found in many sources of fruits, vegetables, and grains, and provide a substrate to be utilized by the gut microbiota of the intestinal tract to produce SCFAs that potentially improve energy homeostasis. Fibers such as resistant starch, β-glucan, wheat fiber, pectin, inulin-type fructans, and soluble corn fiber have all shown encouraging results at improving energy homeostasis and glucose metabolism, possibly via alterations to the gut microbiome. While some fibers such as high-amylose maize or pectin have mixed results regarding the actual effectiveness of the treatment, diets including wheat dextrin, β-glucan, and inulin/oligofructose have extensive evidence suggesting beneficial effects on energy and glucose homeostasis, with inulin and oligofructose being the most promising (see Table 2 for summary of human research). This may be due to the solubility of the fiber, making it more accessible to be fermented by the gut microbiota, or it may be due to other factors such as viscosity or the substrates these fibers are broken down into during fermentation [125]. In human trials, β-glucan, wheat dextrin, and inulin-type fructans have repeatedly resulted in improved body composition and parameters of glucose homeostasis in obese, diabetic, and healthy individuals [21,47,51,55,62,65,66,72,100,102,111]. However, studies examining the metabolic improvements following high-amylose maize, pectin, or soluble corn fiber supplementation are not as conclusive [22,33,37,43]. Because of these apparent differences in fiber supplements, there is a need for rigorous, randomized controlled clinical trials with strict dietary control to minimize the potential confounding effect of overall dietary fiber consumption to confirm efficacy. This would allow for reliable comparison across fiber types, with as little variability in the type or amount of fiber consumed as possible. Additionally, more well-designed human studies are needed with direct comparisons of different fiber supplementations within the same study design to comprehensively compare the metabolic impact among fibers. Along these lines, combination fiber supplementation with two or more types of fiber requires further investigation to determine if combination supplementation has a potential additive or synergistic beneficial effect on metabolic homeostasis. Current research on this topic is limited, although one study demonstrated that mice fed an assorted fiber diet containing resistant starch type 2 and 4, short-chain fructo-oligosaccharides, inulin, and pectin had improvements in energy and glucose homeostasis but these did not reach or exceed the effect of inulin supplementation alone [23]. This may be a result of antagonistic effects of soluble and insoluble fibers; insoluble fibers can increase speed of digestion and cause diarrhea, decreasing the amount of time the soluble fiber remains in the gut for fermentation by bacteria. However, given most humans ingest a variety of different dietary fibers, further research in this area is crucially needed.

As summarized throughout the review, the beneficial effects of various fibers are likely complex and heterogenous, and requires future investigation to potentially create more targeted therapeutic approaches to maximize the beneficial effects of dietary fiber (Figure 1). Nonetheless, studies suggest the gut microbiota is required, given the inability of the host to metabolize the fiber sources. Interestingly, recent work has established that the success of certain therapies to improve metabolic homeostasis depend on the baseline gut microbiome composition [13]. Indeed, this is likely the case with the success of various dietary fibers, as how efficiently the carbohydrate source is broken down will depend on the bacteria present. As such, several studies have found that differences in baseline gut microbiome of rodents affected the success of dietary fiber supplementation [126,127]. For example, genetically identical mice with different microbiota compositions receiving the same fiber-supplemented diet had varying metabolic outcomes, suggesting that supplementation may need to be unique to the individual based on the individual’s microbiome composition [126]. Further, human studies have detailed associations between baseline species richness, as well as specific bacterial taxa (i.e., *Firmicutes*), and the metabolic and/or gut microbiota response to a fiber treatment (reviewed by Hughes et al. [128]). As such, future research could lead to the development of ‘precision nutrition’ that predicts the most efficacious fiber dependent upon an individual’s gut microbiome.

In addition to tailoring the dietary fiber based on the host, scientists are uncovering and developing novel fibers that could potentially increase the effectiveness of treatment. For example, chitin-glucan is a copolymer of chitin bonded to β-glucan. In preliminary studies, it was demonstrated to improve metabolic homeostasis in diet-induced obese mice and caused bacterial shifts and increased fecal SCFAs during 3 week supplementation in humans [28,47,52,55]. Additionally, studies are beginning to examine the ability to transform insoluble fibers to more soluble ones to improve digestion [129] or to try and replace polysaccharide chains directly with SCFAs to site-deliver SCFAs to the colon instead of relying on bacterial fermentation [130]. Furthermore, newly composed synthetic fibers such as Gum Arabic, also known as Acacia Gum, have been shown to decrease BMI and adiposity in humans with type 2 diabetes [127], however more research is necessary to determine mechanistic differences with synthetic fibers. Lastly, foods themselves could be altered by replacing sugars with various dietary fibers to lower the glycemic index of foods while maintaining palatability and taste, which has promise to alter mainstream ingredient formulation [108]. Taken together, given the extensive evidence of the beneficial effects of dietary fibers in improving metabolic homeostasis, future work is warranted to develop novel therapies incorporating their benefits.

## Figures and Tables

**Table 1:** Properties and dietary sources of each fiber type.

Fiber	Properties			Dietary Sources
	Solubility	Viscosity	Fermentation	
High-amylose maize	Mixed	High	Easily fermented	Corn
β-glucan	High	High	Easily fermented	Oat, barley, mushrooms, seaweed
Wheat bran	Insoluble	Low	Poorly fermented	Whole-wheat grains
Wheat dextrin	High	Low	Easily fermented	Whole-wheat grains
Pectin	High	Low	Easily fermented	Apples, carrots, citrus
Inulin-type fructans	Dependent on source	Low	Easily fermented	Chicory root, garlic, onions, leeks
Soluble corn fiber	High	Low	Easily fermented	Corn

**Table 2:** Summary of human fiber research with outcomes involving energy homeostasis, glucose homeostasis, lipid metabolism, and/or inflammation. Downward pointing arrow indicates significant decrease in outcome in intervention group (compared to control or baseline); upward pointing arrow indicates significant increase in outcome in intervention group (compared to control or baseline).

Author (Year)	Fiber Type	Treatment	Duration	Participants	Outcome	Effect
Dainty (2016)	High-amylose maize	bagel with 60% of the wheat flour replaced with Hi-Maize 260	57 d	Men and women with overweight/obesity and increased risk of T2D	Body weight	ns
					Fasting blood glucose	ns
					Plasma glucose C_MAX_	ns
					3-h glucose iAUC	ns
					Fasting insulin	↓
					Serum insulin C_MAX_	ns
					3-h insulin iAUC	↓
					HOMA-IR	↓
					HOMA-%B	↓↓
					HOMA-%S	↑
Maki (2012)	High-amylose maize	high-amylose corn starch containing ~60% RS or a control starch containing no RS, 15 or 30 g/day high-amylose maize	4 wk	Healthy men and women with waist circumference ≥89.0 cm for females or ≥102.0 cm for males	Body weight	ns
					HOMA-%S, men	↑
					acute insulin response to i.v. glucose	ns
					glucose effectiveness	ns
					HOMA-%B	ns
					HOMA-%S, women	ns
Petersen (2018)	High-amylose maize	45 g/d of high-amylose maize or an isocaloric amount of amylopectin	12 wk	Overweight men and women with prediabetes	Body weight	ns
					Fat mass	ns
					Visceral adipose tissue	ns
					HbA1C	↓
					Fasting blood glucose	ns
					Fasting insulin	ns
					Glucose AUC (3 h mixed meal tolerance test)	ns
					Insulin AUC (3 h mixed meal tolerance test)	ns
					Insulin sensitivity	ns
					TNF-a	↓
Bodinham (2014)	High-amylose maize	67 g Hi-maize 260 (60% RS) or 27 ;g Amioca	12 wk	Men and women with T2D	Body weight	ns
					Body mass index	ns
					Fat mass	ns
					HbA1C	ns
					Fasting blood glucose	ns
					Fasting insulin	ns
					HOMA %S	ns
					HOMA %B	ns
					NEFA	↑↑
					TG	↓
					Total cholesterol	ns
					HDL-cholesterol	ns
					LDL-cholesterol	ns
					GLP-1	↑
					TNF-a	↑
					IL-6	ns
					Postprandial AUC0–120 min	↓
Wolever (2020)	β-glucan	cream of rice or instant-oatmeal plus either 3 g oat-bran (2 g oat β-glucan), 10 g oat-bran ^$^ (4 g oat β-glucan)	single meal	Healthy men and women	Glucose iAUC0–2	↓
					Glucose iAUC2–3	ns
					Glucose iAUC0–3	↓
					Glucose peak rise	↓
					Insulin iAUC0–2	↓
					Insulin iAUC2–3	ns
					Insulin iAUC0–3	↓
					Insulin peak rise	↓
					PYY iAUC0–3	ns
					Ghrelin net AUC0–3	ns
Nicolosi (1999)	β-glucan	7.5 g yeast-derived β-glucan fiber consumed twice daily	8 wk	Males with obesity	Body weight	ns
					Body mass index	ns
					Total cholesterol	↓
					LDL cholesterol	ns
					HDL cholesterol	ns
					Total cholesterol: HDL cholesterol	↓
Shimizu (2008)	β-glucan	test diet containing 50% pearl barley and 50% rice (3.5 g β-glucan), or placebo diet containing 100% rice (0.06 g β-glucan) consumed twice daily	12 wk	Males	Total cholesterol	↓
					LDL cholesterol	↓
					Body weight (change from baseline)	↓↓↓
					Waist circumference	ns
					Visceral fat area	↓↓
					Subcutaneous fat area	ns
Aoe (2017)	β-glucan	mixture of rice and high β-glucan barley (test group, 4.4 g/d) or β-glucan–free barley (placebo group)	12 wk	Individuals with waist circumference ≥85 cm for men or ≥90 cm for women and body mass index ≥ 24 kg/m^2^	Body weight	ns*↓↓ ª
					Body mass index	ns*↓↓ ª
					Waist circumference	↓↓*ns ª
					Visceral fat area	↓↓*ns ª
					Subcutaneous fat area	ns *ª
					Total cholesterol	ns *ª
					Triacylglycerol	ns *ª
					HDL cholesterol	ns *ª
					LDL cholesterol	ns *ª
					HbA1C	↑↑*ns ª
					Insulin	ns *ª
					NEFA	↓↓*ns ª
					Glucose	↑*ns ª
Vitaglione (2009)	β-glucan	bread containing 3% β-glucan or control bread containing no β-glucan	single test meal, 4×	Healthy men and women	Hunger AUC_0–60 min_	ns
					Hunger AUC_60–180 min_	↓
					Fullness AUC_0–60 min_	ns
					Fullness AUC_60–180 min_	↑
					Satiety AUC_0–60 min_	ns
					Satiety Fullness AUC_60–180 min_	↑
					Energy intake	↓
					Glucose AUC 3 h following meal	↓
					Plasma ghrelin AUC_0–60 min_	ns
					Plasma ghrelin AUC_60–180 min_	↓
					Plasma PYY	↑
Mosikanon (2016)	β-glucan	477 mg/capsule of β-glucan or rice flour as placebo	6 wk (measurements taken at 2 and 6 weeks, indicated by ^2, 6^)	Men and women with overweight/obesity	Body weight	ns^2,6^
					Body mass index	ns^2,6^
					Waist circumference	↑^2^↑^6^
					Triglycerides	ns^2,6^
					Total cholesterol	ns^2,6^
					HDL cholesterol	ns^2,6^
					LDL cholesterol	ns^2,6^
					TNF-a	↓^2^ns^6^
					IL-6	ns^2^↓^6^
					IL-10	↑^2^↑^6^
Aliasgharzadeh (2015)	Wheat dextrin	supplement of 10 g/d of resistant dextrin and a similar amount of maltodextrin as placebo	8 wk	Women with T2D	Body weight	↓*ns ª
					Body mass index	↓*ns ª
					Energy consumption	↓*ns ª
					Fasting blood glucose	ns *ª
					Fasting insulin	↓ *ª
					HbA1C	ns *ª
					HOMA-IR	↓ *ª
					TNF-a	↓ *ª
					IL-6	↓ *ª
					Endotoxin	↓ *ª
Capomolla (2019)	Pectin	low ^#^ (650 mg) or high ^$^ (1300 mg) dose bergamot juice extract containing 8% pectin	90 d	Individuals with metabolic syndrome	Body weight	ns^#^↓^$^
					Body mass index	↓↓↓^#^↓↓↓^$^
					Total cholesterol	↓↓↓^#^↓↓↓^$^
					LDL cholesterol	↓↓↓^#^↓↓↓^$^
					HDL cholesterol	↑↑^#^↑↑↑^$^
					Triglycerides	↓↓↓^#^↓↓↓^$^
					Fasting blood glucose	↓↓↓^#^↓↓↓^$^
					HOMA-IR	↓↓^#^↓↓↓^$^
Schwartz (1988)	Pectin	20 g/day apple pectin powder in a muffin	4 wk	Men and women with T2D	Gastric emptying	↑
					Glucose iAUC 3 h following test meal	↑↑
					Body weight	ns
					Plasma glucagon	ns
					Gastrin	ns
Wilms (2019)	Pectin	15 g/day sugar beet derived pectin or placebo containing 15 g/day maltodextrin	4 wk	Healthy young adults (18–40 years of age) and healthy elderly (65–75 years of age)	Gastroduodenal permeability	ns
					Small intestinal permeability	ns
					Colonic permeability	ns
					Whole gut permeability	ns
					Junctional complex related gene expression	ns
					Defense and immune related genes	ns
van der Beek (2018)	Inulin	high-fat milkshake containing 24 g inulin of which 0.5 g was U-13C-inulin or placebo containing 24 g maltodextrin	Single dose followed by 5 d washout	Healthy men with overweight/obesity	Fat oxidation iAUC_0–3 h_	↑
					Fat oxidation iAUC_3–7 h_	ns
					Fat oxidation iAUC_0–7 h_	ns
					Carbohydrate oxidation iAUC_0–3 h_	↓
					Carbohydrate oxidation iAUC_3–7 h_	ns
					Carbohydrate oxidation iAUC_0–7 h_	ns
					Energy expenditure	ns
					Free fatty acids iAUC_0–3 h_	↑
					Free fatty acids iAUC_3–7 h_	ns
					Free fatty acids iAUC_0–7 h_	ns
					Triglycerides	ns
					Glucose iAUC_0–3 h_	↓
					Glucose iAUC_3–7 h_	ns
					Glucose iAUC_0–7 h_	ns
					Insulin iAUC_0–3 h_	↓
					Insulin iAUC_3–7 h_	↓
					Insulin iAUC_0–7 h_	↓
					GLP-1	ns
					PYY	ns
Guess (2015)	Inulin	30 g/day inulin or cellulose placebo	18 wk, outcomes assessed at week 9 and 18 (indicated by ^9, 18^)	Men and women with prediabetes	Δ Body weight	ns^9^↓^18^
					Δ Body fat	↓↓^9^ns^18^
					Intrahepatocellular lipid	↓^9, 18^
					Intramyocellular lipid in the soleus muscle	↓↓↓^9^↓^18^
					Δ Fasting plasma glucose	↓↓^9^ns^18^
					Plasma glucose AUC (following mixed meal test)	ns^9, 18^
					Fasting insulin	ns^9, 18^
					Plasma insulin AUC (following mixed meal test)	ns^9, 18^
					HOMA-IR	ns^9, 18^
					Matsuda index	ns^9, 18^
					Plasma GLP-1 AUC (following mixed meal test)	↓^9^↓↓↓^18^
Zhang (2015)	Oligofructose	diet based on whole grains, traditional Chinese medicinal foods, and prebiotics with 3 ready-to-consume food products containing fructo-oligosaccharides and oligoisomaltoses	90 d, measurements taken at 30, 60, 90 d (indicated by ^30, 60, 90^)	Morbidly obese children with Prader-Willis syndrome (PWS) or simple obesity (SO)	Body weight (PWS)	↓↓^30^↓↓^60^↓↓^90^
					Body weight (SO)	↓↓
					Body mass index (PWS)	↓↓^30^↓↓^60^↓↓^90^
					Body mass index (SO)	↓↓
					Fasting glycemia (PWS)	↓↓^30^↓↓^60^↓↓^90^
					Fasting glycemia (SO)	↓
					OGTT Glucose AUC (PWS)	ns^30^↓↓^60^↓↓^90^
					OGTT Glucose AUC (SO)	ns
					Fasting insulinemia (PWS)	↓↓^30^↓↓^60^↓↓^90^
					Fasting insulinemia (SO)	↓↓
					OGTT Insulin AUC (PWS)	↓↓^30^↓↓^60^↓↓^90^
					OGTT Insulin AUC (SO)	↓
					HbA1C (PWS)	↓↓^30^↓↓^60^↓↓^90^
					HbA1C (SO)	↓↓
					Total cholesterol (PWS)	↓↓^30^↓↓^60^↓↓^90^
					Total cholesterol (SO)	↓↓
					Triglycerides (PWS)	↓↓^30^↓↓^60^↓^90^
					Triglycerides (SO)	↓↓
					LDL Cholesterol (PWS)	↓↓^30^↓↓^60^↓↓^90^
					LDL Cholesterol (SO)	↓↓
					Free fatty acids (PWS)	↑^30^ns^60^↑^90^
					Free fatty acids (SO)	↑↑
					IL-6 (PWS)	ns^30,60,90^
					IL-6 (SO)	↓
					LPS binding protein (PWS)	↓↓^30^ns^60,90^
					LPS binding protein (SO)	↓↓
Parnell (2017)	Oligofructose	oligofructose supplement (21 g per day) or an isocaloric maltodextrin placebo	12 wk	Adults with overweight/obesity	Body weight	↓
					Fat mass	↓
					IL-6	ns
					TNF-a	ns
					MCP-1	ns
					Resistin	ns
					PAI-1	↓
					LPS	↓
Parnell (2009)	Oligofructose	oligofructose supplement (21 g per day) or an isocaloric maltodextrin placebo	12 wk	Adults with overweight/obesity	Body weight	↓
					Fat mass	↓
					Trunk fat	↓
					Ghrelin	↓
					PYY	↑
					GLP-1	ns
					Energy intake	↓
					Insulin tAUC 6 h meal tolerance test	ns
					Glucose tAUC 6 h meal tolerance test	ns
Hume (2017)	Oligofructose	8 g per day of oligofructose-enriched inulin or placebo	16 wk	Male and female children with overweight or obesity (BMI ≥85th percentile)	Energy intake	ns
					GIP	ns
					Ghrelin	↑
					Insulin	ns
					GLP-1	ns
					PYY	ns
					Body mass index	ns
Pol (2018)	Oligofructose	bar containing 16 g oligofructose twice a day	12 wk	Men and women with overweight/obesity	Body weight	ns
					Waist circumference	ns
					Fat mass	ns
					Body fat %	ns
					Total energy intake	ns
					Hunger	ns
					Fullness	ns
Lightowler (2018)	Oligofructose	yogurt drink containing 5.6 g oligofructose from chicory instead of sucrose was compared with a control yogurt drink	Single dose	Healthy men and women	Glucose iAUC120min	↓
					Glucose peak	↓
					Insulin iAUC120min	↓↓
					Insulin peak	ns
Lightowler (2018)	Inulin	fruit jelly containing 13 g inulin from chicory instead of sucrose was compared with a control fruit jelly	Single dose	Healthy men and women	Glucose iAUC120min	↓
					Glucose peak	↓↓
					Insulin iAUC120min	↓↓↓
					Insulin peak	↓↓↓
Tan (2020)	Soluble corn fiber	four test meals (two solid and two beverages), 25 g total carbohydrates consisting of either glucose or glutinous rice and the other 25 g carbohydrates consisting of either soluble corn fiber or maltodextrin	Single dose	Males with a body mass index between 18.5–30.0 kg/m2	Glucose iAUC130min (drink)	↓
					Glucose iAUC130min (rice)	↓
					Insulin iAUC130min (drink)	↓
					Insulin iAUC130min (rice)	↓↓
Costabile (2017)	Soluble corn fiber	L. rhamnosus GG-PB12 combined with soluble corn fiber, L. rhamnosus GG combined with soluble corn fiber, soluble corn fiber alone, or placebo (results shown only for soluble corn fiber vs. placebo)	3 wk	Healthy, elderly men and women	Total cholesterol	ns
					HDL cholesterol	ns
					LDL cholesterol	ns
					Triglycerides	ns
					Non-esterified fatty acids	ns
					Glucose	ns
					Total cholesterol: HDL cholesterol	ns
					IL-6	↓
					IL-8	ns
					Body weight	ns
					Body mass index	ns
					Waist circumference	ns
Konings (2014)	Soluble corn fiber	test meal with 54.6 g soluble corn fiber and two control diets (full energetic and isoenergetic)	Single meal	Overweight men and women	Postprandial glucose AUC	ns
					Postprandial insulin AUC	↓
					Non-esterified fatty acid AUC	↑
					Triglyceride AUC	ns
					Energy expenditure (24 h)	ns
Babiker	Gum arabic	30 g of powdered gum arabic or 5 g of placebo	3 mo	Men and women with T2D	Body weight	↓↓
					Waist circumference	ns
					Triglycerides	ns
					HDL cholesterol	↑
					Waist to hip ratio	ns
					Body mass index	↓↓
					Body adiposity index	↓↓
					Visceral adiposity index	↓
					Deep abdominal adipose tissue	ns

Ns = no statistical difference.↓ *p* ≤ 0.05, ↓↓ *p* ≤ 0.01, ↓↓↓ *p* ≤ 0.001; ↑ *p* ≤ 0.05, ↑↑ *p* ≤ 0.01, ↑↑↑ *p* ≤ 0.001; * change from baseline, ª change from control group. ^$^ represent results from the low and high dose treatment.

## Data Availability

Not applicable.
